# Fatty Acid Binding Proteins FABP9 and FABP10 Participate in Antibacterial Responses in Chinese Mitten Crab, *Eriocheir sinensis*

**DOI:** 10.1371/journal.pone.0054053

**Published:** 2013-01-24

**Authors:** Lin Cheng, Xing-Kun Jin, Wei-Wei Li, Shuang Li, Xiao-Nv Guo, Juan Wang, Ya-Nan Gong, Lin He, Qun Wang

**Affiliations:** School of Life Science, East China Normal University, Shanghai, China; National Cancer Institute, NIH, United States of America

## Abstract

Invertebrates rely solely on the innate immune system for defense against pathogens and other stimuli. Fatty acid binding proteins (FABP), members of the lipid binding proteins superfamily, play a crucial role in fatty acid transport and lipid metabolism and are also involved in gene expression induced by fatty acids. In the vertebrate immune system, FABP is involved in inflammation regulated by fatty acids through its interaction with peroxidase proliferator activate receptors (PPARs). However, the immune functions of FABP in invertebrates are not well characterized. For this reason, we investigated the immune functionality of two fatty acid binding proteins, Es-FABP9 and Es-FABP10, following lipopolysaccharide (LPS) challenge in the Chinese mitten crab (*Eriocheir sinensis*). An obvious variation in the expression of Es-FABP9 and Es-FABP10 mRNA in *E. sinensis* was observed in hepatopancreas, gills, and hemocytes post-LPS challenge. Recombinant proteins rEs-FABP9 and rEs-FABP10 exhibited distinct bacterial binding activity and bacterial agglutination activity against *Escherichia coli* and *Staphylococcus aureus*. Furthermore, bacterial growth inhibition assays demonstrated that rEs-FABP9 responds positively to the growth inhibition of *Vibrio parahaemolyticuss* and *S. aureus*, while rEs-FABP10 responds positively to the growth inhibition of *Aeromonas hydrophila* and *Bacillus subtilis*. Coating of agarose beads with recombinant rEs-FABP9 and rEs-FABP10 dramatically enhanced encapsulation of the beads by crab hemocytes in vitro. In conclusion, the data presented here demonstrate the participation of these two lipid metabolism-related proteins in the innate immune system of *E. sinensis*.

## Introduction

Fatty acid binding proteins (FABPs) belong to a family of small proteins (14–15 kDa), which are principally located in the cytosol and characterized by the ability to bind non-covalently and with high affinity to hydrophobic ligands, such as retinol, retinoic acid, bile salts and pigments, especially long-chain fatty acids (LCFAs) [1], [2]. FABPs are implicated in general lipid metabolism, acting as intracellular transporters of hydrophobic metabolic intermediates and as carriers of lipids between membranes [2], [3]. To date, 12 FABP isoforms have been identified in vertebrates [4]. Isoform expression among multiple tissues and differences in tissue distribution among FABP orthologs have resulted in the implementation of a numeric nomenclature. Based on this system of nomenclature, FABP9 and FABP10 correspond to T-FABP and L-FABP, respectively [5], [6].

The physiological functions of FABPs include, but are not limited to, the uptake and utilization of fatty acids, intracellular targeting of fatty acids to specific organelles and metabolic pathways, and the protection of cellular structures from the detergent effects of fatty acids [2], [3], [7]. Fatty acids have diverse roles in all cells, functioning as energy sources, cell membrane components, and signal molecules. Fatty acids are now also thought to have a particularly important immune function [8], especially in inflammation reactions [9]. The first FABP was reported in 1972 [10], so vertebrates have been studied in detail for about four decades up to now. However, only fewer have been identified in invertebrates, invertebrate FABPs were first discovered in the desert locust *S. gregaria*, and approximately 30 have been identified in this species now [1], [6]. Although several invertebrate FABPs have been found in Crustacea and Insecta [1], some reports suggest that FABPs are associated with immunity [11].

In vertebrates, FABPs have been studied in detail, and evidences indicate that FABPs are involved in immune reactions, such as; recombinant FABP from parasites such as *Schistosoma japonicum* are used as a vaccine in susceptible animals [12], [13]. In mice challenged with *P. aeruginosa*, FABP had been shown to be involved in the negative correlation between cell surface expression of the cystic fibrosis transmembrane conductance regulator (CFTR) and NF-κB mediated inflammatory signaling [14]. L-FABP expression was significantly altered after injection of pathogen and lack of adaptive immunity, played roles in cellular antioxidant defense by binding to PUFAs (polyunsaturated fatty acids) [15]–[18]. E-FABP possibly through regulation of TNF-α production to play a role in the host defense against bacterial infection, and effectively as a marker for dendritic cells and it may be participated in antigen capture through combination with fatty acids [19]–[21]. aP2, the adipocyte fatty acid binding protein, regulated allergic airway inflammation and inflammatory responsiveness, and had a significant role in against atherosclerosis responses [14], [18], [22]–[24]. The interaction between FABPs and PPARs involved in immune reactions has been confirmed in numerous studies [6], [8], [24].

In invertebrates, several FABPs have been identified in Platyhelminths, Nematoda, Acarida, Crustacea and Insecta, and recent reports have indicated that FABP improves memory and provides a novel role in regulating memory consolidation in *Drosophila* [25]. However, few reports suggest that FABPs is associated with immunity [1]. For example, differential expression of L-FABP and H-FABP was observed in *Fenneropenaeus chinensis* challenged with white spot syndrome virus (WSSV) and Vibrio [26]. Suppression subtractive hybridization results showed upregulated expression of FABP in the hepatopancreas of WSSV resistant and susceptible shrimp innate immunity [27]. Expression of FABP was increased sharply after WSSV infection of *Procambarus clarkii* [28], in the presence of pl-FABP (*Pacifastacus leniusculus*), which supported the hypothesis that a RA (retinoic acid) signaling pathway is likely to be present in crustacea, and that this pathway might be more active during stress [29]. In Chinese white shrimp, *Fenneropenaeus chinensis*, Fc-FABP was detected mainly in the hepatopancreas and the expression level increased after challenge with WSSV, Fc-FABP was downregulated by *V. anguillarum* challenge. The protein also had bacterial binding activity and it could be speculated that the downregulation of Fc-FABP in shrimp results in high levels of ROS [11]. In studies of parasitized *P. xylostella* larvae, approximately 50 proteins, including FABP, were differentially expressed every 48 h [30], thus implicated FABP in the immune response against infection, although the exact mechanism remains to be elucidated.

Invertebrates do not possess an adaptive immune system due to the absence of vital elements such as antibodies, lymphocytes and immunological memory [31]. However, invertebrates such as crustaceans and insects are capable of generating a highly effective innate immune system including cellular and humoral components under the selective pressure imposed by infectious microorganisms [2]. The first line of host defense against pathogenic organisms and other foreign materials involves innate immune recognition mechanisms [32], which is mediated by innate recognition receptors and immune effectors [33]. Due to the structural variation of infectious pathogens [34], [35], host immunity evolves a wide range of different recognition receptors and immune effectors to recognize the corresponding PAMPs (pathogen associated molecular patterns) including lipopolysaccharide (LPS), peptidoglycans (PGNs) and β-1-3-glucans [36], and to stimulate a series of inflammatory responses. A recent report showed that FABPs bind bacteria in Chinese white shrimp [11], for this reason, the function of FABPs in invertebrate immunity requires further investigation.

The Chinese mitten crab (*Eriocheir sinensis*), which is a commercially important aquaculture species in South-East Asia, has been cultured in ponds, reservoirs and lakes since the 1990s and now is widely harvested in China [37]. Recent study have indicated that FABP as an important factor in immune responses [8]. To prove the point, in this study, we investigated the expression profile of Es-FABP9 (testis FABP) and Es-FABP10 (liver basic FABP) [38]–[40], following a challenge with LPS, which is a major component of the Gram-negative bacterial cell wall, and purified the recombinant proteins rEs-FABP9 and rEs-FABP10. Furthermore we detected the binding activity with bacteria and the bacterial growth inhibitory ability of the two proteins, simultaneously observed bacterial encapsulation of rEs-FABP9 and rEs-FABP10 coated Ni-NTA agarose beads by microscopy.

## Materials and Methods

### 2.1. Animal challenged with LPS and sample preparation

Healthy adult Chinese mitten crabs (n = 140; 100±20 g wet weight) were collected from the Tongchuan aquatic product market in Shanghai, China, and acclimated for one week at 20–25°C in filtered, aerated freshwater. For LPS stimulation, 120 crabs were divided equally into two groups: experimental group crabs were injected into the arthrodial membrane of the last pair of walking legs with approximately 100 ml of LPS (Sigma-Aldrich, L2630) resuspended (500 mg/ml) in PBS, while control group with 100 ml PBS (pH = 7.4). Five crabs were randomly selected at each time interval of 0 (as blank control), 2, 4, 8, 12, 24, 48 and 72 h post injection, and subsequently placed in an ice bath for 1–2 min until each was lightly anesthetized. Hemolymph was draw from the hemocoel in arthrodial membrane of the last pair of walking legs using a syringe (approximately 2.0 ml per crab) with an equal volume of anticoagulant solution (glucose: 2.05 g, citrate: 0.8 g, NaCl: 0.42 g, double distilled water: add to 100 ml) added, and centrifuged at 800 *g* at 4°C to isolate hemocytes. The other tissues (hepatopancreas, gills) were harvested, snap frozen in liquid nitrogen, and stored at −80°C, after the addition of 1 ml Trizol reagent (Invitrogen, CA, USA) for subsequent RNA extraction. Except the 40 crabs were sacrificed for tissue collection respectively, experimental group had 11 individuals death, and then control group had 0 individuals death until 72 h post challenged.

### 2.2. Total RNA isolation

Total RNA was extracted from *E. sinensis* tissues using Trizol® reagent (RNA Extraction Kit, Invitrogen, CA, USA) according to the manufacturer's protocol. The total RNA concentration and quality were estimated using spectrophotometry at an absorbance at 260 nm and agarose-gel electrophoresis, respectively. For quantitative real-time PCR (qRT-PCR) expression analysis, total RNA (4 µg) was reverse transcribed using the SYBR® Premix Ex Taq™ Real-time PCR Kit (TaKaRa, Dalian, China).

### 2.3. Transcription analysis post LPS challenged by qRT-PCR

The mRNA expressions of Es-FABP9 and Es-FABP10 were measured by qRT-PCR. Briefly, total RNA was isolated from hemocytes, heart, hepatopancreas, gills, stomach, muscles, and intestines of unchallenged crabs and from hemocytes, hepatopancreas and gills of LPS-challenged crabs. qRT-PCR was conducted using the CFX96TM Real-Time System (Bio-Rad). Gene-specific primers (Table 1) were designed based on the cloned cDNA open reading frames (ORFs) of Es-FABP9 and Es-FABP10 to produce fragments of 411 bp and 393 bp, respectively. Samples were run in triplicate and normalized to the control gene β-actin (Table 1) [41]. Es-FABP9 and Es-FABP10 expression levels were calculated by the 2^−ΔΔ^Ct comparative CT method [42]. qRT-PCR amplification reactions were carried out in a final volume of 25 µl, which contained 12.5 µl 2X SYBR Premix Ex Taq (TaKaRa), 0.5 µl (500 ng/µl) diluted cDNA template, 11.0 µl PCR-Grade water (RNase free, TaKaRa), and 0.5 µl (10 µM) of each primer, and the samples were run in triplicate. PCR conditions were as follows: 95°C for 30 s, followed by 40 cycles of 95°C, and a 0.5°C/5 s incremental increase from 60°C to 95°C (30 s per cycle). Resulting data were analyzed using the CFX Manager™ software (Version 1.0).

**Table 1:** Primer sequences.

Primers name	Sequences (5′-3′)
***qRT-PCR***	
EsFABP9-F	ATGGACGCAATGTGAA
EsFABP9-R	CGAACACGCACAATCC
EsFABP10-F	CCCTCGCTGTTTCTACCA
EsFABP10-R	GCCGTGGTCTTGATGACGATGT
β-actin-F	CTCCTGCTTGCTGATCCACATC
β-actin-R	GCATCCACGAGACCACTTACA
***Sequencing***	
T7 promoter	TAATACGACTCACTATAGG
T7 terminator	CACCGCTGAGCAATAACTAGC

### 2.4. Construction of the expression plasmid

Primers designed for amplification of the ORF sequences of Es-FABP9 and Es-FABP10 included restriction enzyme cutting sites in the 5′ terminal of the primers. Sequences were amplified using the following primers: Es-FABP9-F: 5′-GGAATTCCATATGATGGCCAAGATCGTTGGAAAGTA-3′; Es-FABP9-R: 5′- CCGCTCGAGCTAGTCTAGACGCTTGTACACGC-3′; Es-FABP10-F: 5′-GGAATTCCATATGATGTCCATCACCGGGAAATACGT-3′;Es-FABP10-R:5′-CCGCTCGAGCTACTGGCGGGAGTAGATCCTCTTG-3′. pET28a (p)-Es-FABP9 and pET28a (p)-Es-FABP10 were generated by combining the ORF of Es-FABP9 or Es-FABP10 with the *NdeI* and *XhoI* sites for cloning into pET28a (p) with His-taq. These constructed plasmids were transformed into *E. coli* BL21-DE5 competent cells for recombinant expression.

### 2.5. Expression and purification of recombinant protein

Overnight cultures of transformants (1 ml) were added to 50 ml kanamycin-containing Luria-Bertani (LB) broth for largescale culture. When the OD_600_ value reached 0.6, isopropyl-b-D-1-thiogalactopyranoside (IPTG) was added into the culture (1 mM final concentration) for induction of Es-FABP9 and Es-FABP10 expression. After culturing overnight at 37°C, 50 ml the recombinant bacteria were collected by centrifugation (6000×*g*) for 5 min. The pellets were resuspended in 8 ml of guanosine Lysis Buffer and ultrasonication. The 8 ml lysate was then added to a prepared Purification Column and purified by affinity chromatography using His-binding resin chromatography (Invitrogen, Carlsbad, USA) following the manufacturer's instructions under native conditions. After 30–60 min in order to keep the resin suspended in the lysate solution, the target protein with His-taq is binding to the resin of Purification Column, then low speed centrifugation (800×*g*) and wash the resin with 8 ml Native Wash Buffer for four times, and finally elute the protein with 8–12 ml Native Elution Buffer. Routine protein estimation was conducted using the Bradford method, using bovine serum albumin (BSA) as the standard.

### 2.6. Bacteria binding assay by Western blot

Two Gram-positive bacteria (*staphylococcus aureus* and *Bacillus subtilis*) and two Gram-negative bacteria (*Vibrio parahaemolyticus* and *Aeromonas hydrophila*) were used to test binding activity of the recombinant proteins. Bacteria were cultured in 5 ml LB medium overnight and were pelleted by centrifugation (6000×*g*) for 5 min, washed twice with TBS, and then thoroughly resuspended in 2 ml TBS (OD_600_ approximately 1.0). Purified recombinant rEs-FABP9 and rEs-FABP10 (0.1 mg/ml; 500 µl) were incubated with rotation with 500 µl microorganisms (2×10^7^ cells/ml) in TBS for 1 h at 37°C. The mixed microorganisms were pelleted (6000×*g*, 5 min), washed four times with TBS, and then eluted with 10% SDS (20 µl). Eluents were resuspended in SDS-PAGE loading buffer and separated by 12% SDS-PAGE. Recombinant rEs-FABP9 and rEs-FABP10 proteins were detected by Western blot using specific His-tag antibodies [11].

### 2.7. Bacteria growth inhibitory assay

The growth curves of rEs-FABP9 in response to *V. parahaemolyticus* and *S. aureus*, and of rEs-FABP10 in response to *A. hydrophila* and *B. subtilis* were analyzed as follows: the four types of bacteria were cultured overnight at 30°C in 10 ml LB broth and adjusted to an OD_600_ value of approximately 1.0 with sterile LB broth. Different final concentrations of rEs-FABP9 were added to 2 ml cultures of *V. parahaemolyticus* or *S. aureus*, or rEs-FABP10 was added to and *A. hydrophila* or *B. subtilis*, and cultured for different times. Each sample was incubated with aeration at 200 rpm and the OD_600_ was measured every 2 h [43].

### 2.8. Bacterial agglutination assay

The bacterial agglutination activity of rEs-FABP9 and rEs-FABP10 was assessed using previously described methods [44], [45]. The Gram-negative bacteria *E. coli* (DH5α) and Gram-positive *S. aureus* were cultivated in LB medium overnight and centrifuged (6000×*g*) for 5 min [44]. The bacteria were washed and resuspended in TBS (50 mM Tris-HCl, 100 mM NaCl, pH 7.5) and adjusted to the OD_600_ value of approximately 4.0. Bacterial suspensions (10 µl) were then added to 50 µl of recombinant rEs-FABP9 and rEs-FABP10, and 50 µl TBS solution was used as a control. The mixtures were incubated at room temperature (1–2 h). To assess the dose-dependency of agglutination, different doses of proteins were incubated with the bacteria under the conditions described above. The reactions were observed by bright light microscopy.

### 2.9. In vitro cellular adhesion assay

The reactions of the two recombinant proteins in the presence of the hemocytes were investigated as previously described [46]. In this study, Ni-NTA agarose beads were washed three times and equilibrated in TBS, before rEs-FABP9, rEs-FABP10 and rTrx (recombinant Thioredoxin) as a control protein were added and incubated with shaking at room temperature for 1 h. Protein-coated beads were washed with TBS four times, and resuspended with TBS. Hemocytes were collected from Chinese mitten crabs using a sterile syringe and simultaneously diluted in anticoagulant. Hemocytes were added to each group of the protein-coated beads. Hemocytes were incubated with the recombinant protein-coated agarose beads at 18°C for 6 h and 24 h. The final reactions were observed by light microscopy [47].

### 2.10. Bioinformatics analysis and statistical analysis

Sequences similarity analysis was performed with BLAST program (http://www.ncbi.nlm.nih.gov/) and multiple sequence alignment was conducted using ClustalW2. Signal sequence and motif prediction were performed using SMART (http://smart.embl-heidelberg.de/). MEGA 5.0 was used to produce the NJ-phylogenetic tree for analysis of FABP9 and FABP10 with representative invertebrates and vertebrates sequences from Protein Blast results [48].

Statistical analysis was performed using SPSS software (Ver11.0). The datas are represented as the mean ± standard error (S.E.). Statistical significance was determined by one-way ANOVA [49] and post-hoc Duncan multiple range tests. Significance was set at P<0.05.

## Results

### 3.1. Bioinformatics analysis of Es-FABP9 and Es-FABP10

Multiple sequence alignment of FABPs involved in immunity by ClustalW2 revealed significant sequence similarity among vertebrate and invertebrate FABPs (Fig. S1). Furthermore, Es-FABP9 exhibited significant homology at the amino acid level with *Procambarus clarkii*, *Penaeus monodon* and *Metapenaeus ensis*, while Es-FABP10 exhibited greater similarity with *Fenneropenaeus chinensis*. The observed sequence similarity among invertebrate FABPs indicated a high degree of sequence and domain conservation.

**Figure 1:**
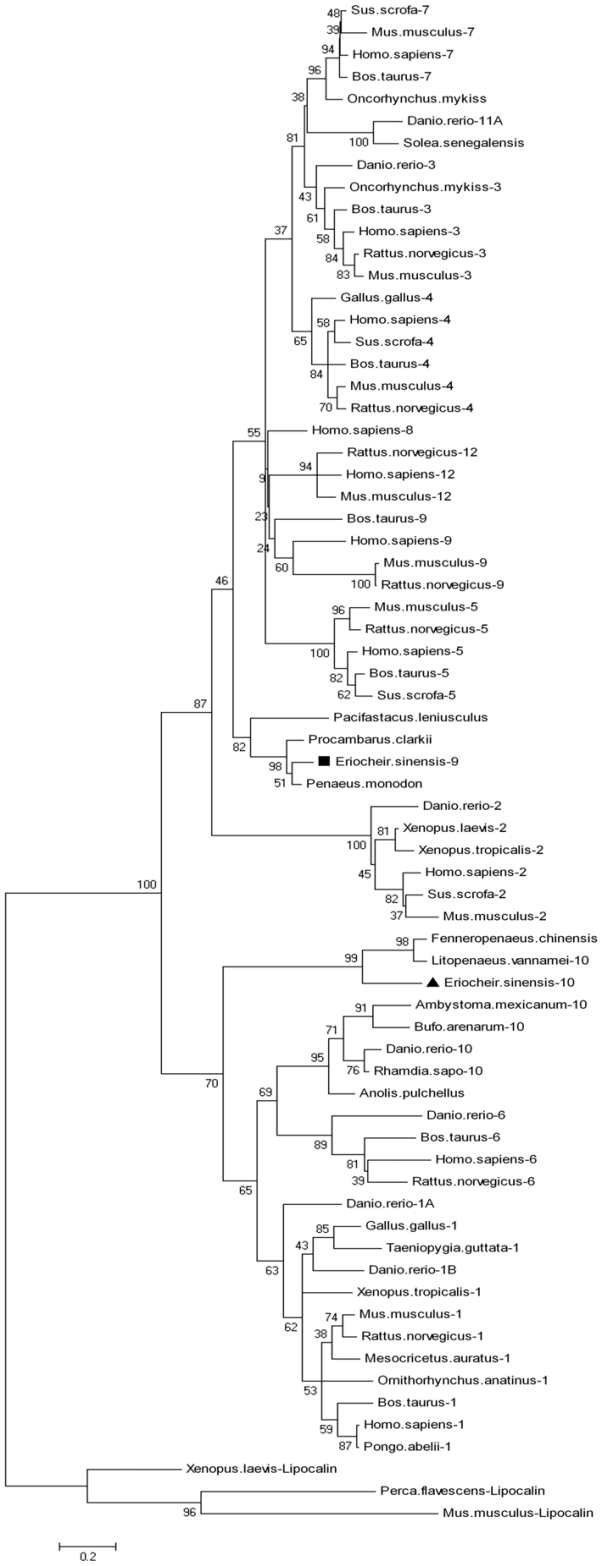
Phylogenetic analysis of *Eriocheir.sinensis* FABP with other known FABPs of sequences from GenBank. NJ tree was produced with MEGA 5.0. *Ambystoma.mexicanum*-10 (P81400.2); *Bufo arenarum*-10 (P83409.2); *Danio rerio*-1B (AAZ08576.1); *Danio rerio*-1A (NP_001038177.1); *Danio rerio*-3 (NP_694493.1); *Danio rerio*-6 (ACD37360.1); *Danio rerio*-10 (AAI64928.1); *Danio rerio*-11A (NP_001004682.1); *Fenneropenaeus chinensis* (ACU82845.1); *Gallus gallus*-1 (NP_989523.1); *Gallus gallus*-4 (NP_989621.1); *Homo sapiens*-1 (NP_001434.1); *Homo sapiens*-4 (NP_001433.1); *Homo sapiens*-5 (NP_001435.1); *Homo sapiens*-6 (AAH22489.1); *Homo sapiens*-7 (CAG33338.1); *Homo sapiens*-8 (NP_002668.1); *Homo sapiens*-12 (NP_001098751.1); *Mus musculus*-1 (NP_059095.1); *Mus musculus*-2 (AAS00550.1); *Mus musculus*-7 (CAJ18607.1); *Mus musculus*-9 (NP_035728.2); *Mus musculus*-12 (NP_083586.1); *Oncorhynchus mykiss*-3 (NP_001118185.1); *Oncorhynchus mykiss* (AEW44189.1); *Pongo abelii*-1 (NP_001125017.1); *Rattus norvegicus*-3 (NP_077076.1); *Rattus norvegicus*-6 (NP_058794.1); *Rattus norvegicus*-9 (NP_074045.1); *Rattus norvegicus*-12 (NP_001128086.1); *Rhamdia sapo*-10 (P80856.2); Sus scrofa-2 (NP_001026950.1); *Sus scrofa*-4 (NP_001002817.1); *Sus scrofa*-5 (NP_001034835.1); *Sus scrofa*-7 (AAY17257.1); *Solea senegalensis* (CAM58515.1); *Taeniopygia guttata*-1 (XP_002188068.1); *Xenopus laevis*-2 (NP_001079346.1); *Penaeus monodon* (ABE77154.1); *Pacifastacus leniusculus* (ABE77153.1); *Xenopus tropicalis*-1 (AAI61795.1); *Bos taurus*-1 (DAA24570.1); *Homo sapiens*-2 (AAH69617.1); *Danio rerio*-2 (AAH75970.1); *Xenopus tropicalis*-2 (AAI35506.1); *Homo sapiens*-3 (CAG33148.1); *Bos taurus*-3 (NP_776738.1); *Mus musculus*-3 (NP_034304.1); *Mus musculus*-4 (CAJ18597.1); *Bos taurus*-4 (DAA22709.1); *Rattus norvegicus*-4 (AAH84721.1); *Bos taurus*-5 (DAA22652.1); *Mus musculus*-5 (NP_034764.1); *Rattus norvegicus*-5 (NP_665885.1); *Bos taurus*-6 (DAA27219.1); *Bos taurus*-7 (DAA26338.1); *Bos taurus*-9 (DAA22692.1); *Homo sapiens*-9 (NP_001073995.1); *Ornithorhynchus anatinus*-1 (XP_001510550); *Litopenaeus vannamei*-10 (ABD65306.1); *Mesocricetus auratus*-1 (AAV33399.1); *Rattus norvegicus*-1 (NP_036688.1); *Procambarus clarkii* (ADY80038); *Anolis pulchellus* (AAA68960.1); *Xenopus laevis*-Lipocalin (NP_001081513.1); *Mus musculus*-Lipocalin (NP_084235.1); *Perca flavescens*-Lipocalin (ACO82027.1).

Phylogenetic analysis by MEGA 5.0 software showed that the FABPs form a large family (Fig. 1), which can be divided into two branches. Branch1 includes FABP7s, FABP3s, FABP4s, FABP12s, FABP9s, FABP5s and FABP2s, while branch2 contains FABP10s, FABP6s and FABP1s. Es-FABP9 together with *Procambarus clarkia* and *Penaeus monodon* FABPs were belong to a subbranch in branch1; *Litopenaeus vannamei* FABP10, *Fenneropenaeus chinensis* FABP and Es-FABP10 are clustered into a subbranch in branch2. The results were consistent with the evolutionary relationship between the zebrafish fabp11 gene and the tetrapod FABP4, FABP5, FABP8 and FABP9 genes [50].

### 3.2. Temporal expression of Es-FABP9 and Es-FABP10 post LPS immune challenge

**Figure 2:**
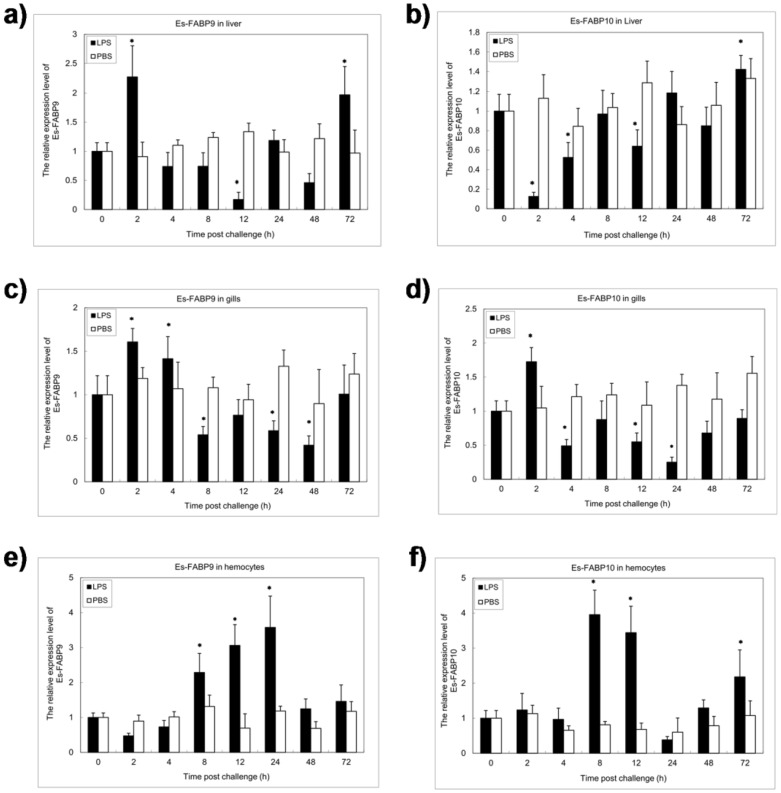
Quantitative real-time PCR analysis of Es-FABP9 and Es-FABP10 in response to LPS immune challenged. Es-FABP9 and Es-FABP10 expression have significantly alteration in comparison to the control which post injection with LPS at 2, 4, 6, 8, 12, 24, 48 and 72 h in varying tissues. (a), Es-FABP9 expression in hepatopancreas (liver); (b), Es-FABP10 expression in hepatopancreas; (c), Es-FABP9 expression in gills; (d), Es-FABP10 expression in gills; (e), Es-FABP9 expression in hemocytes; (f), Es-FABP10 expression in hemocytes.

The expression of Es-FABP9 and Es-FABP10 following LPS challenge was analyzed in hepatopancreas, gills and hemocytes which as the primary immune tissues [51]. Es-FABP9 was dramatically upregulated in the hepatopancreas at 2 h post challenge with LPS, and was downregulated at 12 h (Fig. 2a). However, Es-FABP10 was markedly downregulated in the hepatopancreas at 2 h post LPS challenge, and was upregulated at 4 h (Fig. 2b). Maximum expression of both proteins was observed at 72 h. Statistical analysis showed that there was significant difference in Es-FABP9 and EsFABP-10 transcript levels in the hepatopancreas post LPS challenge. In gill tissue, the relative expression of Es-FABP9 post LPS challenge was upregulated at 2 h and 4 h, and downregulated at 8 h (Fig. 2c). Es-FABP10 expression was upregulated at 2 h and downregulated rapidly at 4 h (Fig. 2d). After 4 h, the expression levels of both Es-FABP9 and Es-FABP10 fluctuated until normal levels were recovered. In hemocytes, no obvious change in Es-FABP9 expression was detected at 2 h and 4 h, although expression was suddenly upregulated at 8 h (Fig. 2e). Es-FABP10 exhibited a similar expression pattern (Fig. 2f), and expression of both successively increased to maximal expression detected at 24 h, followed by decreased levels of expression.

### 3.3. Expression and purification rEs-FABP9 and rEs-FABP10 recombinant protein

**Figure 3:**
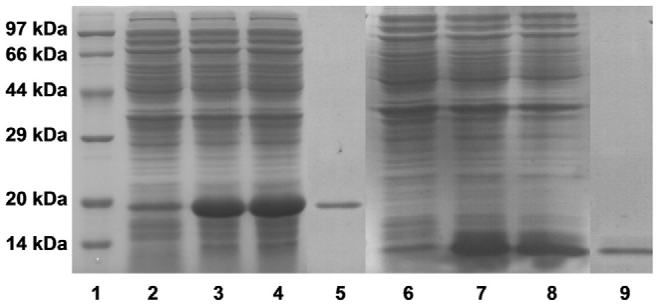
SDS-PAGE results of recombinant expression and purification of rEs-FABP9 and rEs-FABP10 in *E. coli*. Lane 1, protein standard; Lane 2, total proteins of *E. coli* transfected with pET28a-Es-FABP9, without induction; Lane 3, pellets of *E. coli* transfected with pET28a-Es-FABP9, induced with IPTG; Lane 4, supernatants of *E. coli* transfected with pET28a-Es-ABP9, induced with IPTG; Lane 5, recombinant rEs-FABP9 purified by His-Bind resin chromatography; Lane 6, total proteins of *E. coli* transfected with pET28a-Es-FABP10, without induction; Lane 7, pellets of *E. coli* transfected with pET28a-Es-FABP10, induced with IPTG; Lane 8, supernatants of *E. coli* transfected with pET28a-Es-ABP10, induced with IPTG; Lane 9, recombinant rEs-FABP10 purified by His-Bind resin chromatography.

Recombinant rEs-FABP9 and rEs-FABP10 were expressed in *E. coli* (DH5α), purified as described in section 2.6 and analyzed by SDS-PAGE. rEs-FABP9 and rEs-FABP10 have predicted molecular weight of approximately 15 kDa and 13 kDa. However, both proteins were expressed as fusion protein with His-tag at the C-terminal. These extra amino acids increased the molecular mass of the expressed target proteins to approximately 19 kDa and 14 kDa respectively (Fig. 3).

### 3.4. Binding activity of rEs-FABP9 and rEs-FABP10 towards bacteria

**Figure 4:**
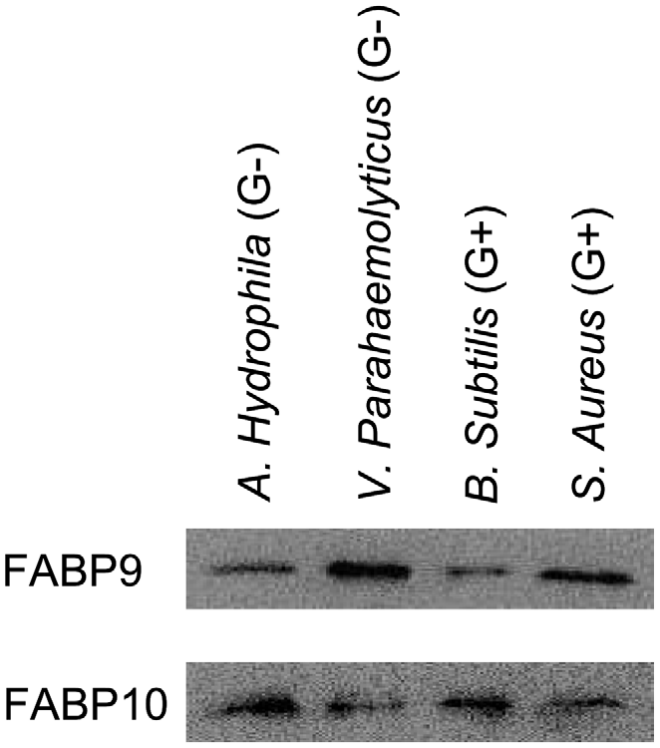
Binding of the microorganisms by rEs-FABP9 and rEs-FABP10. Living microbial strains, two strains of Gram-negative bacteria (G^−^) and two strains of Gram-positive bacteria (G^+^) were incubated with the proteins. Western blot analysis using His-tag antibody and the development used ECL coloration kit (KangWei, Beijing, China).

Western blot analysis showed that both rEs-FABP9 and rEs-FABP10 were capable of binding directly to microorganisms, and the results were consistent with our preliminary experiments used by *Escherichia Coli*. The results indicated that rEs-FABP9 binds four bacterial strains, but *V. parahaemolyticus* and *S. aureus* were bound with the greatest efficiency. However, rEs-FABP10 was also shown to bind all four bacterial strains, and *A. hydrophila* and *B. subtilis* were bound with the greatest efficiency. These observations indicated distinct binding activities of the two recombinant proteins (Fig. 4).

### 3.5. Antibacterial activity of rEs-FABP9 and rEs-FABP10

**Figure 5:**
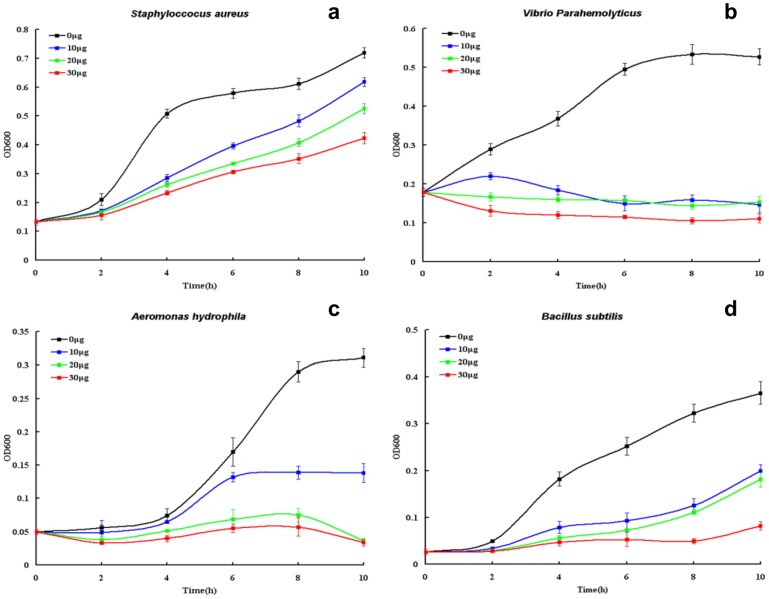
Bacterial agglutination activity assay towards Gram-negative bacteria *E. coli* and Gram-positive bacteria *S. aureus*. *E. coli* was in (a, b, e, f, i, j) and *S. aureus* was in (c, d, g, h, k, l). (a–d) were incubated with rTrx as a control, (e–h) were incubated with rEs-FABP9 and (i–l) were incubated with rEs-FABP10. The proteins' final quantity of (a–l) are different as followed. (a, c): rTrx, 5 µg, (b, d): rTrx, 10 µg; (e, g): rEs-FABP9, 5 µg, (f, h): rEs-FABP9, 10 µg; (i, k): rEs-FABP10, 5 µg, (j, l): rEs-FABP10, 10 µg.

The results of Western blotting analysis of binding activity were used to select the appropriate bacteria for investigation of the effect of concentration on the antimicrobial activity of rEs-FABP9 and rEs-FABP10. Compared with the rTrx, the antimicrobial activity of rEs-FABP9 against *V. parahaemolyticus* and *S. aureus* was dose-dependent with 30 µg/ml rEs-FABP9 strongly suppressing microbial growth (Fig. 5). However, microbial growth was weakly suppressed by 10 µg/ml rEs-FABP9. The antimicrobial activity of rEs-FABP10 against *A. hydrophila* and *B. subtilis* was dose-dependent with 30 µg/ml EsFABP10 strongly suppressing microbial growth, but 10 µg/ml EsFABP10 mediating only weak suppression.

### 3.6. Bacterial agglutination

**Figure 6:**
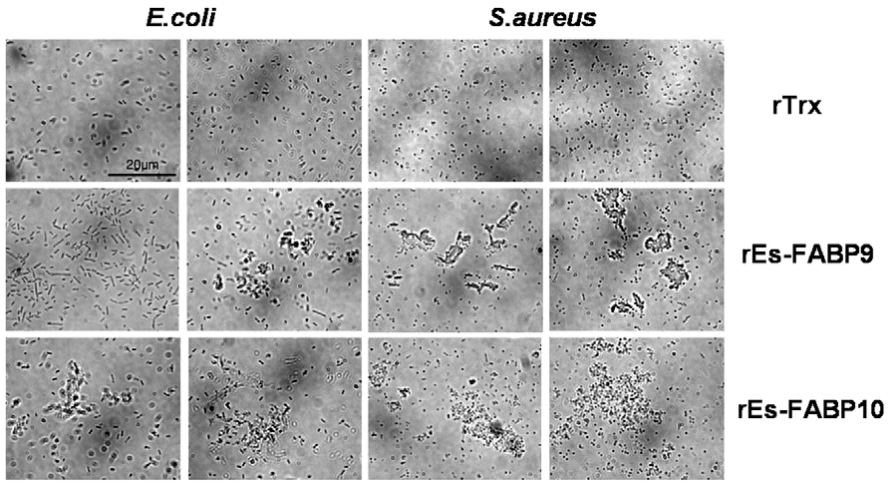
rEs-FABPs possessed antimicrobial activity. Growth suppression tests of TBS and rEs-FABPs against Gram-negative and Gram-positive bacteria. OD_600_ values were measured every 2 h after starting the cultures, and the growth curves were drawn. (a, b): *S. aureus* (G^+^) and *V. parahaemolyticus* (G^−^) mixed with rEs-FABP9 respectively, (c, d): *A. hydrophila* (G^−^) and *B. subtilis* (G^+^) mixed with rEs-FABP10 respectively. The data are the average ±SD of three independent cultures. ---, 0 µg; ---, 10 µg; ---, 20 µg; ---, 30 µg.

Gram-negative *E. coli* and Gram-positive *S. aureus* were used to test the bacterial agglutination activity of the rEs-FABP9 and rEs-FABP10 proteins, in comparison with TBS as a control. Microscopic observation showed that maximum bacterial agglutination activity was achieved in the presence of 0.1 mg/ml recombinant rEs-FABP9 and rEs-FABP10, although rEs-FABP9 exhibited weaker bacterial agglutination activity than rEs-FABP10 against *E. coli* and *S. aureus*. No agglutination was observed in TBS controls (Fig. 6).

### 3.7. In vitro cellular adhesion

**Figure 7:**
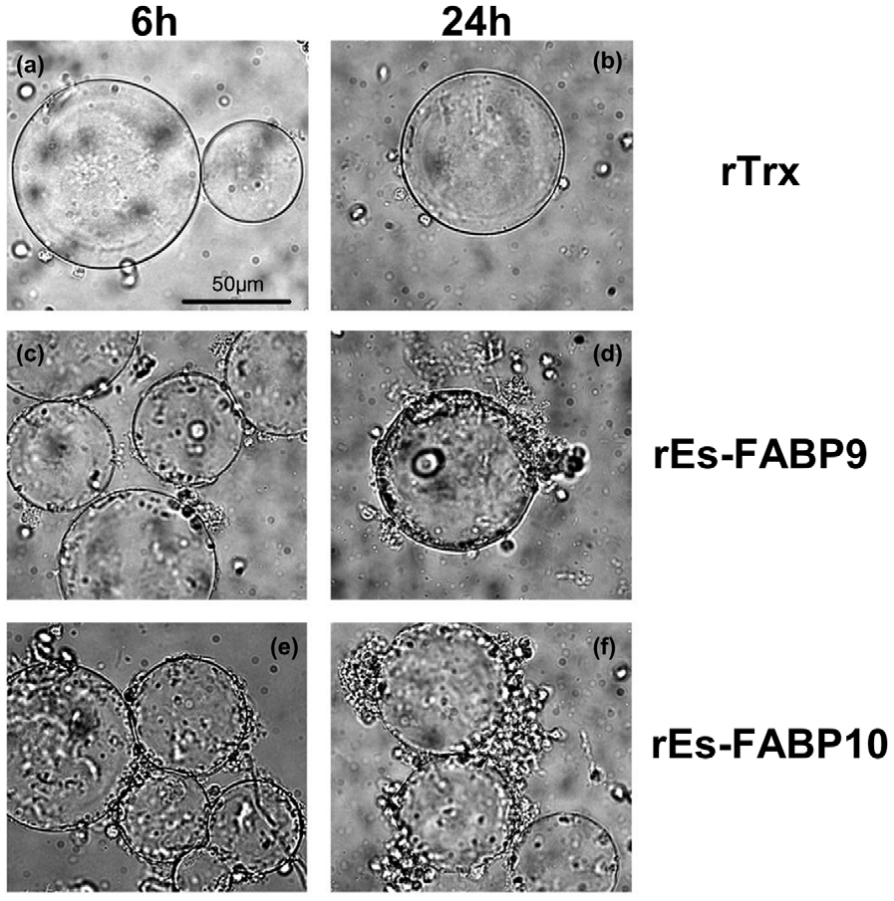
rEs-FABPs promote encapsulation by hemocytes. Coating with rEs-FABP9 and rEs-FABP10 and rTrx (as a control) were incubated with hemocytes for 6 h and 24 h respectively at room temperature. Encapsulation of the protein-coated beads was observed by light microscopy: (a, b) rTrx-coated beads were encapsulated by hemocytes, (c, d) rEs-FABP9-coated beads were encapsulated by hemocytes and (e, f) rEs-FABP10-coated beads were encapsulated by hemocytes. Scale bar, 50 µm.

In order to investigate whether rEs-FABP9 and rEs-FABP10 promote cellular adhesion, we used protein-coated agarose beads and hemocytes in vitro encapsulation assays (Fig. 7). Vitro cellular adhesion assay was performed in different wells for statistic analysis. We observed approximately 75% of the beads coated with rEs-FABP9 and rEs-FABP10 was encapsulated by hemocytes at 6 h, while fewer beads (26%) coated with rTrx were encapsulated. After 24 h, most of the beads coated with rEs-FABP9 (79%) and rEs-FABP10 (81%) were encapsulated by hemocytes, while fewer beads coated with rTrx (24%) were encapsulated.

## Discussion

Although many studies have focused on the classification, structure and function of vertebrate FABPs, these proteins remain uncharacterized in many invertebrate species. FABPs participate mainly in the uptake and utilization of fatty acids, intracellular targeting of fatty acids to specific organelles and some metabolic pathway, and the protection of cellular structures [52]. In vertebrates, FABPs play an important role in the pathway mediated by fatty acids and are involved in immunity [53]. However, the function of FABPs in the immune system of *E. sinensis* remains largely unknown.

In our study, temporal expression patterns of Es-FABP9 and Es-FABP10 in the primary immune tissues (hepatopancreas, gills and hemocytes) of Chinese mitten crabs were investigated following LPS challenge. This two genes were both immune-stimulated separately which may be identified as anti-bacteria characterization. In hepatopancreas, the transcription level of Es-FABP9 was instantly upregulated and downregulated later, while the Es-FABP10 was significantly downregulated at 2 h post LPS challenge, and then upregulated at 4 h. In some shrimps, such as *Fenneropenaeus chinensis* and *Litopenaeus vannamei*, FABP mRNAs were similarly upregulated in the hepatopancreas induced by WSSV [11], [27]. In gills, almost identical variation in Es-FABP9 and Es-FABP10 expression was observed, that were both rapidly upregulated and dramatically downregulated later, which might due to the most sensitive defense mechanism of gills as the first barrier against external environment [54]. In the consideration of decrease of circulating hemocyte counts, that could be a consequence of hemocytes immobilization in the gills, which resulted in the Es-FABPs highly expression in gills, as Lectins in Chinese mitten crab [55]. In hemocytes, the transcript levels of Es-FABP9 and Es-FABP10 were both sharply upregulated at 8 h. This not only confined to *E. sinensis*, in *Procambarus clarkia*, the expression of FABP was found to be upregulated in hemocytes induced by WSSV through the suppression subtractive hybridization and cDNA microarrays [28]. These results were in agreement with the theory that hemocytes from the tissue initially recruited after challenge with microbial polysaccharides, and decrease the rate of apoptosis, indicating that more cells are directed along differentiation pathways instead of undergoing apoptosis [56], [57].

The evidences speculated that different functions of Es-FABP9 and Es-FABP10 in immune tissues based on the mRNA detection by quantitive PCR after LPS challenge were not sufficient. Aiming to further investigated the specific immune activity of Es-FABP9 and Es-FABP10, we obtained the recombinant fusion protein rEs-FABP9 and rEs-FABP10 via prokaryotic expression system, and conducted a series of biological experiments in vitro. Bacteria binding assay showed that rEs-FABP9 and rEs-FABP10 were both able to bind not only Gram-negative but also Gram-positive bacteria, yet with different affinity. In *Fenneropenaeus chinensis*, rFc-FABP binds *vibrio anguillarum* and *S. aureus*, but it binds *V. anguillarum* with greater affinity [11]. This phenomenon might be explained by three differences of binding activity with Gram-positive and Gram-negative bacteria. First, signal sequence and motif prediction analysis of the amino acid sequences and protein domains of Es-FABP9 and Es-FABP10 using SMART software showed that the Es-FABP9's PFAM domain is lipocalin, while the Es-FABP10's PFAM domain is peptidase_C41. Second, the composition of the cell wall differs in the Gram-negative and Gram-positive bacteria. Third, the binding activity of Es-FABP9 and Es-FABP10 with bacteria may depend on the bacterial to which they bind.

The bacteria inhibitory growth curves displayed that the bacteria growth were suspended and even prohibited after different concentration of rEs-FABP9 and rEs-FABP10 added. We observed rEs-FABP9 had more inhibitory activity towards *V. parahaemolyticus* than *S. aureus*, while rEs-FABP10 had more inhibitory activity towards *A. hydrophila* than *B. subtilis*. In the other studies, a kind of C-type lectin AmphiCTL1 from amphioxus could bind and kill *S. aureus* and *S. cerevisiae* in the absence of calcium directly [43], in *Holothuria scabra*, T-antigen binding lectin possessed strong antibacterial activity against both Gram-positive (*Staphylococcus sp.*, *Streptococcus sp.*) and Gram-negative bacteria (*Serratia sp.*, *Proteus sp.*, *Shigella sp.* and *E. coli*) [44], and in *F. chinensis*, a hepatopancreas specific C-type lectin rFc-hsL had antimicrobial activity against *B. megaterium*, *B. thuringiensis*, *S. aureus*, *E. coli* and *K. pneumoniae* [58]. These results consequently illustrated that rEs-FABP9 and rEs-FABP10 probably had the same antibacterial activity with some lectins, which inhibit the bacteria growth via interaction with the bacterial cognate glycan antigen [44].

Based on the results of bacterial agglutination assay, we observed that rEs-FABP9 and rEs-FABP10 have dose-dependent bacterial agglutination activity against *E. coli* and *S. aureus*. Besides, the agglutination activity of rEs-FABP10 was higher than that of rEs-FABP9 both in *E. coli* and *S. aureus*. In *H. scabra*, T-antigen binding lectin HSL regulated agglutination with untreated cells of bacteria such as *E. coli* and *Staphylococcus sp.* obviously [44], in *Haliotis discus*, a novel C-type lectin CLHd could introduce the agglutination of Gram-negative pathogenic *V. alginolyticus* [45], in *Fenneropenaeus chinensis*, agglutination assay revealed Fc-hsL had calcium-dependent agglutinating activity against some Gram-positive and Gram-negative bacteria [58], in *Penaeus japonicus*, a natural lectin PjLec possessed a broadly agglutination activities against both Gram-positive and Gram-negative bacteria, which include two *Vibrio* species and two other strains pathogenic for shrimp [59], and in *Acropora millepora*, the lectin millectin could agglutinate both Gram-positive and Gram-negative bacteria [60]. This result suggests that the rEs-FABP9 and rEs-FABP10 could recognize the cognate glycan antigen on the cell surface that facilitate the bacterial agglutination.

In cellular adhesion assay of the rEs-FABP9 and rEs-FABP10, we observed that both rEs-FABP9 and rEs-FABP10 promoted hemocytes encapsulation in various degrees. In *Manduca sexta*, immulectins (a member of the C-type lectin superfamily) coated in the agarose beads could enhance insect hemocytes cellular encapsulation [46], and in *Chlamys farreri*, an ancient C-type lectin CfLec-2 bound to the surface of scallop hemocytes and arose cellular adhesion to enhance their encapsulation, which revealed CfLec-2 had the function of pathogen recognition and cellular adhesion [47]. According to our observation, rEs-FABP9 activated hemocytes encapsulation and the activity was more effectively at 24 h than at 6 h as well as rEs-FABP10, while there was no obvious encapsulation in the present of rTrx as a control. This phenomenon suggested that Es-FABPs could bind to the surface of hemocytes, activate cellular interaction and play roles in the recognition on cellular level.

In conclusion, our experiments revealed that Es-FABP9 and Es-FABP10 mRNA expression were both altered in different primary immune tissues (hepatopancreas, hemocyte and gills) and different time interval post-LPS challenge. Furthermore, both recombinant proteins possess binding activity, growth inhibitory activity and agglutination activity against bacteria, and mediating simultaneous hemocyte encapsulation in vitro. Our datas indicate that FABP9 and FABP10 are associated with responses to LPS during early infection and may also be involved in general antibacterial defensed by recognize the surface of bacteria and hemocytes in *E. sinensis*. Thus, this study provided new insights into the innate immune system in invertebrates, although, the underlying mechanism of the involvement of FABP9 and FABP10 in these processes requires further investigation.

## Supporting Information

Figure S1 **Multiple sequence alignment of** ***Eriocheir sinensis*** **FABPs with other species FABPs.** FABPs: *Eriocheir sinensis*-10 (ADP05225.1); *Eriocheir sinensis*-9 (ADM64456.1); *Penaeus monodon* (ABE77154); *Pacifastacus leniusculus* (ABE77153); *Metapenaeus ensis* (AAL68638); *Gallus gallus* (NP_989523); *Bos taurus* (NP_787011); *Ornithorhynchus anatinus* (XP_001510550); *Danio rerio* (AAZ08576); *Litopenaeus vannamei-10* (ABD65306); *Mesocricetus auratus*-1 (AAV33399); *Homo sapiens* (AAA52419); *Rattus norvegicus* (NP_036688); *Danio rerio-10* (NP_694492); *Procambarus.clarkii* (ADY80038); *Anolis pulchellus* (AAA68960); *Fenneropenaeus chinensis* (ACU82845.1).
(TIF)
