# Supplementary figures and images for: Fatty Acid Binding Proteins FABP9 and FABP10 Participate in Antibacterial Responses in Chinese Mitten Crab, Eriocheir sinensis
Source: PLoS One. 2013 Jan 24;8(1):e54053. doi: 10.1371/journal.pone.0054053 (PMC3554701; doi:10.1371/journal.pone.0054053)

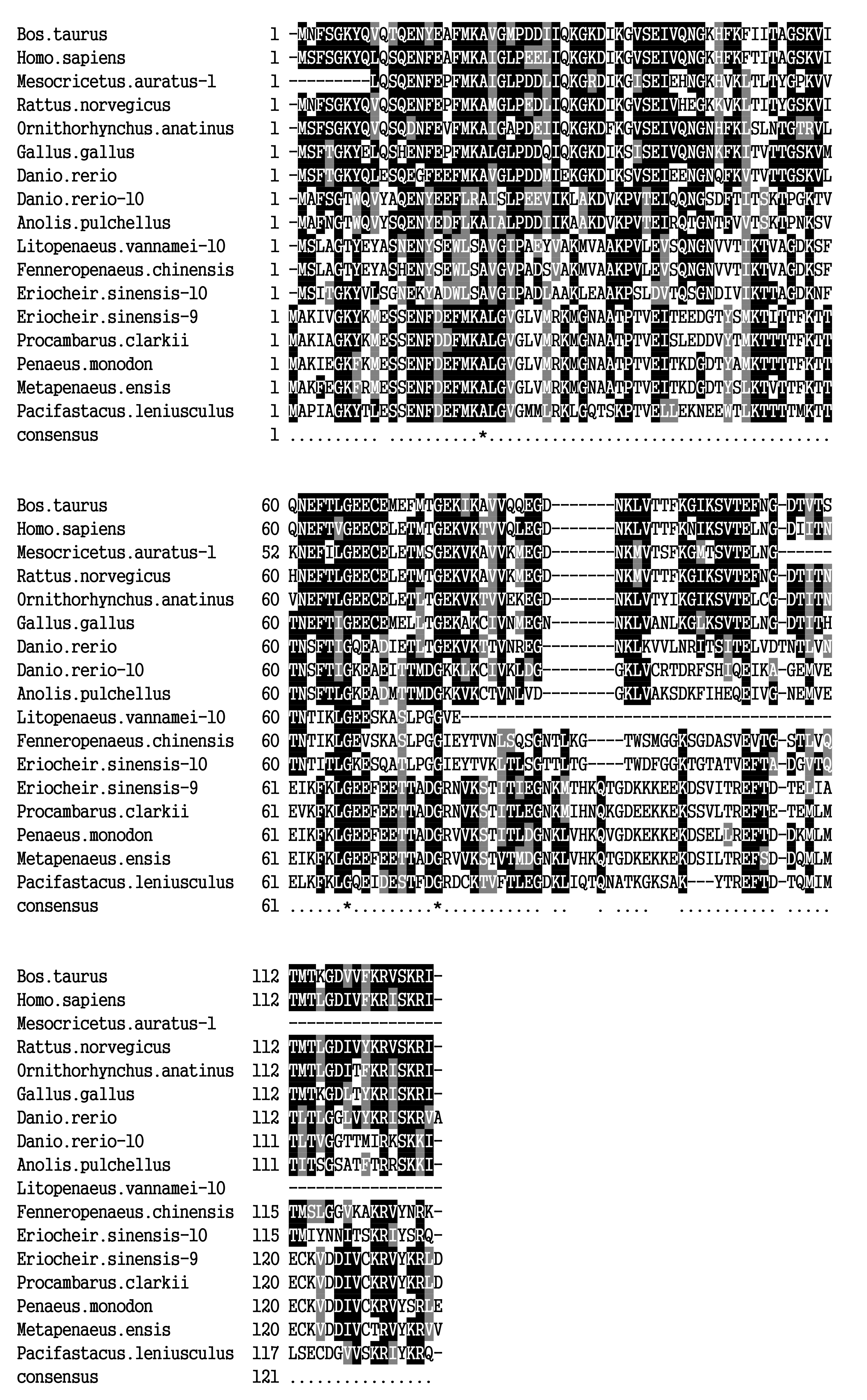

Supplement: Figure S1 — Multiple sequence alignment of Eriocheir sinensis FABPs with other species FABPs. FABPs: Eriocheir sinensis-10 (ADP05225.1); Eriocheir sinensis-9 (ADM64456.1); Penaeus monodon (ABE77154); Pacifastacus leniusculus (ABE77153); Metapenaeus ensis (AAL68638); Gallus gallus (NP_989523); Bos taurus (NP_787011); Ornithorhynchus anatinus (XP_001510550); Danio rerio (AAZ08576); Litopenaeus vannamei-10 (ABD65306); Mesocricetus auratus-1 (AAV33399); Homo sapiens (AAA52419); Rattus norvegicus (NP_036688); Danio rerio-10 (NP_694492); Procambarus.clarkii (ADY80038); Anolis pulchellus (AAA68960); Fenneropenaeus chinensis (ACU82845.1). (TIF) [file pone.0054053.s001.tif]
